# Supplementary material for: Distinct Urinary Proteome Changes Across Estimated Glomerular Filtration Rate Stages in a Cohort of Black South Africans
Source: Int J Mol Sci. 2025 Feb 18;26(4):1740. doi: 10.3390/ijms26041740 (PMC11855517; doi:10.3390/ijms26041740)
Supplement: Supplementary file 1 [file ijms-26-01740-s001.zip › ijms-3422874-supplementary.pdf]

Distinct Urinary Proteome Changes Across Estimated Glomerular Filtration Rate Stages in a Cohort of Black South Africans

Siyabonga Khoza [1,*], Jaya A. George [2,3,4], Previn Naicker [5], Stoyan H. Stoychev [6], Rethabile J. Mokoena [7], Ireshyn S. Govender [5,7,†] and June Fabian [8,9,*,†]

[1] Department of Chemical Pathology, National Health Laboratory Service, Faculty of Health Sciences, University of the Witwatersrand, Johannesburg 2000, South Africa
[2] National Health Laboratory Service, Johannesburg 2192, South Africa
[3] Academic Affairs, Research & Quality Assurance, National Health Laboratory Service, Johannesburg 2000, South Africa
[4] Wits Diagnostic Innovation Hub, University of the Witwatersrand, Johannesburg 2000, South Africa
[5] ReSyn Biosciences, Edenvale 1610, South Africa;
[6] Evosep Biosystems, 5230 Odense, Denmark
[7] Future Production Chemicals, Council for Scientific and Industrial Research, Pretoria 0001, South Africa
[8] Wits Donald Gordon Medical Centre, School of Clinical Medicine, Faculty of Health Sciences, University of the Witwatersrand, Johannesburg 2000, South Africa
[9] South African Medical Research Council/Wits University Rural Public Health and Health Transitions Research Unit (Agincourt), School of Public Health, Faculty of Health Sciences, University of the Witwatersrand, Johannesburg 2000, South Africa
* Correspondence: professor.khoza@wits.ac.za (S.K.); june.fabian@mweb.co.za (J.F.)
† These authors contributed equally to this work.

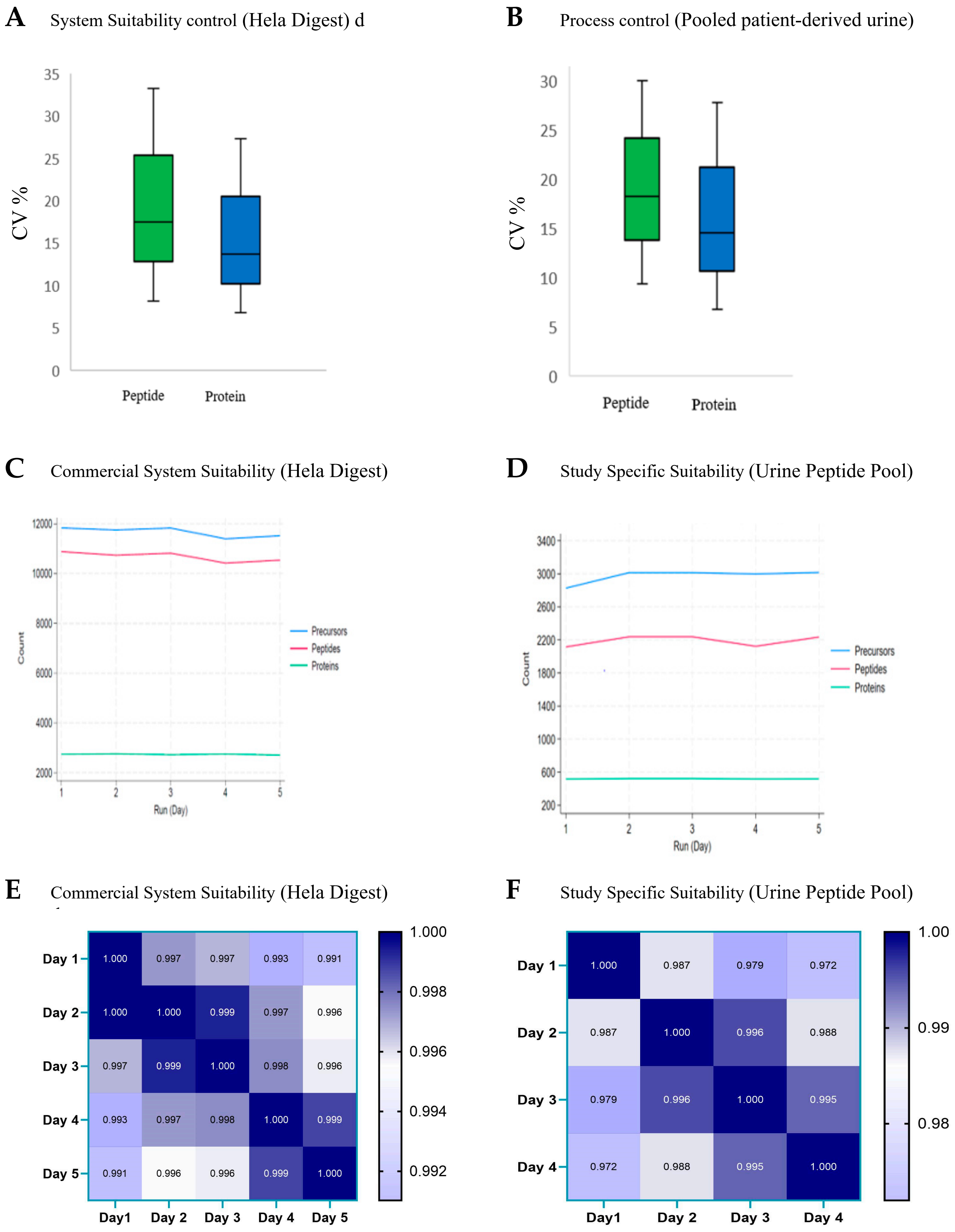

Figure S1. Project suitability quality control (commercial Hela digest and Urine peptide pool). CV, coefficient of variation.

Table S1. List of differentially abundant proteins between Stage G1, Stage G2 and Stage G3 – G5

Protein Name	UniProt Accession Number	Gene Name
Immunoglobulin kappa variable 3D-15	A0A087WSY6	IGKV3D-15
Antithrombin III	P01008	SERPINC1
Beta-2 microglobulin	P61769	B2M
Carbonic anhydrase 1	P00915	CA1
Pro-epidermal growth factor	P01133	EGF
Gelsolin	P06396	GSN
Neural cell adhesion molecule L1-like protein	O00533	CHL1
Beta-1,4-glucuronyltransferase 1	O43505	B4GAT1
Superoxide dismutase [Cu-Zn]	P00441	SOD1
Immunoglobulin kappa variable 3-20	P01619	IGKV3-20
Immunoglobulin lambda variable 1-47	P01700	IGLV1-47
Immunoglobulin heavy constant gamma 3	P01860	IGHG3
Hemopexin	P02790	HPX
Phosphatidylcholine-sterol acyltransferase	P04180	LCAT
Immunoglobulin lambda variable 7-43	P04211	IGLV7-43
Plasma serine protease inhibitor	P05154	SERPINA5
Fatty acid-binding protein, liver	P07148	FABP1
Heat shock protein HSP 90-beta	P08238	HSP90AB1
Immunoglobulin kappa light chain	P0DOX7	P0DOX7
Immunoglobulin lambda-1 light chain	P0DOX8	P0DOX8
Immunoglobulin lambda constant 3	P0DOY3	IGLC3
Heat shock protein 90 beta family member 1	P14625	HSP90B1
Immunoglobulin lambda-like polypeptide 1	P15814	IGLL1
Carboxypeptidase N subunit 2	P22792	CPN2
Insulin-like growth factor-binding protein 6	P24592	IGFBP6
Mannosyl-oligosaccharide 1,2-alpha-mannosidase IA	P33908	MAN1A1
IST1 homolog	P53990	IST1
Desmocollin-2	Q02487	DSC2
Cystatin-M	Q15828	CST6
Glutathione hydrolase 6	Q6P531	GGT6
Peptidase inhibitor 16	Q6UXB8	PI16
Protein FAM3C	Q92520	FAM3C
Neuroserpin	Q99574	SERPINI1
Osteomodulin	Q99983	OMD
Multimerin-2	Q9H8L6	MMRN2
Vacuolar protein sorting-associated protein VTA1 homolog	Q9NP79	VTA1
Sushi domain-containing protein 2	Q9UGT4	SUSD2
Selotransferrin	P02766	TRFE

Stage G1 (eGFR ≥ 90 ml/min/1.73 m²), Stage G2 (eGFR 60–89 ml/min/1.73 m²), and Stage G3 – G5 (eGFR < 60 ml/min/1.73 m²).

Table S2: Pairwise comparisons using Dunn's test with a Bonferroni adjustment indicated that Stage G3–G5 scores

**Protein name**	**Grup 1** **(Stage G1 vs Stage G3–G5)** ***p*-value**	**Group 2** **(Stage G2 vs Stage G3– G5)** ***p*-value**	**Group 3** **(Stage G1 vs Stage G2)** ***p*-value**
IGKV3D-15	0.0002	0.0163	0.0681
SERPINC1	0.0002	0.0009	0.8213
B2M	0.0002	0.0020	0.4830
CA1	0.0001	0.0052	0.1904
EGF	0.0000	0.0004	0.3033
GSN	0.0000	0.0039	0.0701
CHL1	0.0001	0.0021	0.4505
B4GAT1	0.0001	0.0066	0.1512
SOD1	00000	0.0011	0.3270
IGKV3-20	0.0001	0.0149	0.0641
IGLV1-47	0.0007	0.0513	0.0604
IGHG3	0.0001	0.0065	0.0884
HPX	00000	0.0001	0.4859
LCAT	0.0001	0.0034	0.2768
IGLV7-43	0.0001	0.0015	0.3132
SERPINA5	0.0001	0.0072	0.0914
FABP1	0.0018	0.0001	0.3688
HSP90AB1	0.0154	0.0314	1.0000
P0DOX7	0.0002	0.0265	0.0664
P0DOX8	0.0010	0.0756	0.0753
IGLC3	0.0001	0.0088	0.1130
HSP90B1	0.0003	0.0593	**0.0184***
IGLL1	0.0001	0.0094	0.0805
CPN2	0.0000	0.0006	0.4329
IGFBP6	0.0306	0.0001	**0.0092***
MAN1A1	0.0002	0.0266	**0.0372***
IST1	0.0002	0.0035	0.3147
DSC2	0.0001	0.0048	0.1743
CST6	0.0002	0.0269	**0.0403***
GGT6	0.0005	0.1631	**0.0046***
PI16	0.0001	0.0038	0.2207
FAM3C	0.0002	0.0005	1.000
SERPINI1	0.0000	0.0005	0.3873
OMD	0.0009	0.0001	0.6411
MMRN2	0.0007	0.0001	0.7120
VTA1	0.0001	0.0139	0.060
SUSD2	0.0000	0.0074	**0.0019***
TRFE	0.0001	0.0004	1.000

Significant difference between group 1 and group 3 for all proteins. * Significant differences Stage between CKD 1 and CKD 2. CKD 1 (eGFR (G1) ≥ 90 ml/min/1.73 m^2), CKD 2 (eGFR (G2) 60–89 ml/min/1.73 m^2) and CKD 3–5 (eGFR (G3 –G5) < 60 ml/min/1.73 m^2)

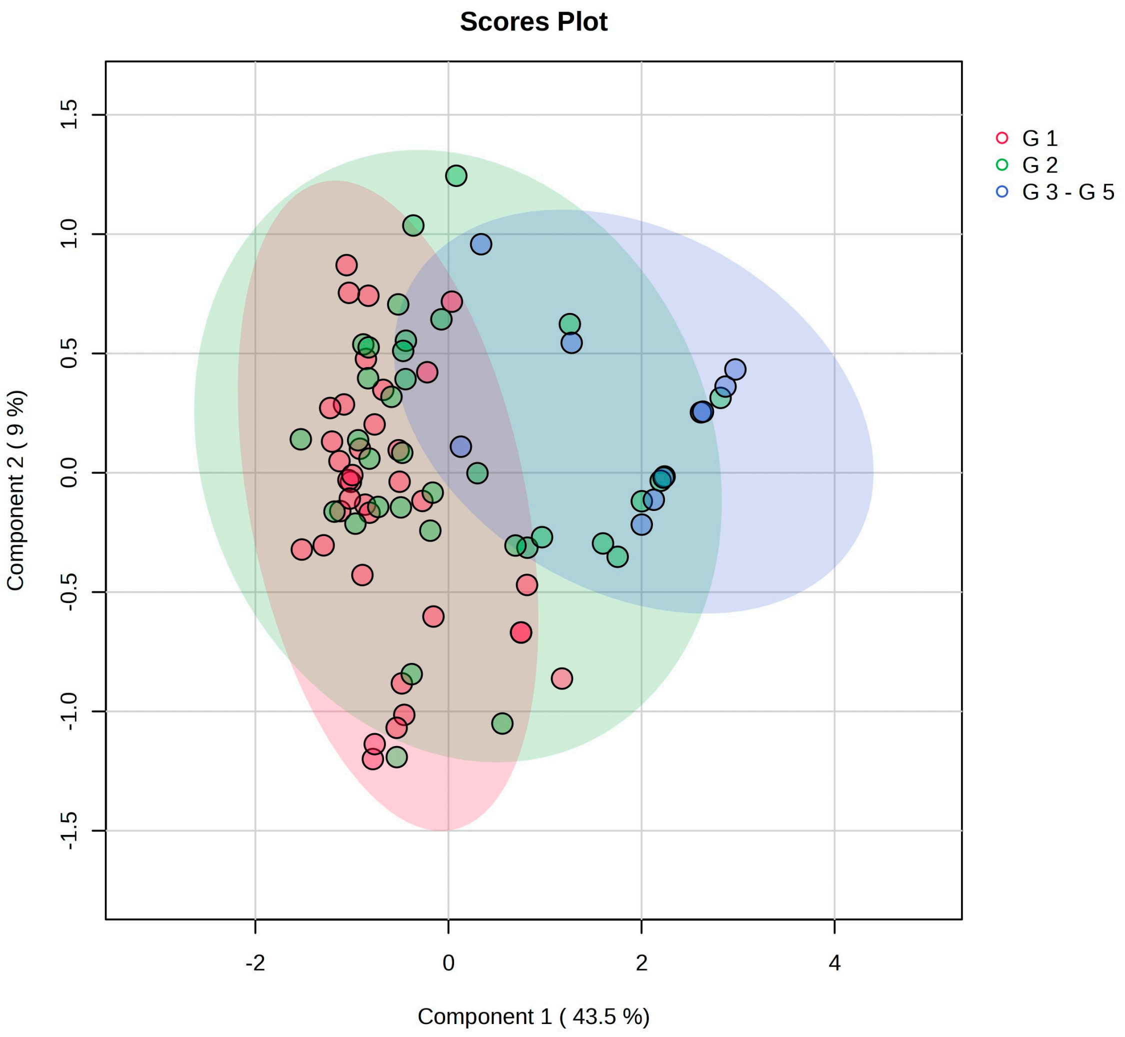

Figure S2. Principal component analysis of 6 differentially abundant proteins for three different groups by eGFR. Stage G1 (eGFR ≥ 90 ml/min/1.73 m²), Stage G2 (eGFR 60–89 ml/min/1.73 m²), and Stage G3 – G5 (eGFR < 60 ml/min/1.73 m²).

Table S3. Correlations of Serum Creatinine, eGFR and uACR with some urinary proteins

Information on protein		Serum creatinine		eGFR		uACR	
**Urinary Proteome**	**Protein Name**	**Spearman's *Rho***	***p*-value**	**Spearman's *Rho***	***p*-value**	**Spearman's *Rho***	***p*-value**
IGKV3D15	Immunoglobulin kappa variable 3D-15	0.31	**0.004**	-0.33	**0.003**	0.30	**0.006**
CST6	Cystatin M	0.43	**<0.001**	-0.31	**0.005**	0.11	0.332
SERPINC1	Antithrombin III	0.22	**0.046**	-0.21	0.064	0.52	**<0.001**
B2M	Beta-2 microglobulin	0.21	0.058	-0.19	0.091	0.39	**<0.001**
CA1	Carbonic anhydrase 1	0.28	**0.013**	-0.31	**0.005**	0.44	**<0.001**
EGF	Pro-epidermal growth factor	-0.25	**0.027**	0.38	**<0.001**	-0.38	**<0.001**
GSN	Gelsolin	0.42	**<0.001**	-0.34	**0.002**	0.34	**0.005**
CHL1	Neural cell adhesion molecule L1-like protein	-0.29	**0.007**	0.29	**0.008**	-0.44	**<0.001**
B4GAT1	Beta-1,4-glucuronyltransferase 1	-0.31	**0.005**	0.37	**<0.001**	-0.31	**0.005**
SOD1	Superoxide dismutase [Cu-Zn]	0.28	**0.011**	-0.23	**0.038**	0.35	**0.002**
HSP90B1	Heat shock protein 90 beta family member 1	-0.37	**<0.001**	0.43	**<0.001**	-0.33	**0.003**
IGKV320	Immunoglobulin kappa variable 3-20	0.32	**0.003**	-0.31	**0.005**	0.29	**0.009**
IGLV147	Immunoglobulin lambda variable 1-47	0.26	**0.018**	-0.25	**0.023**	0.28	**0.012**
IGHG3	Immunoglobulin heavy constant gamma 3	0.19	0.096	-0.30	**0.008**	0.38	**<0.001**
HPX	Hemopexin	0.22	**0.049**	-0.30	**0.007**	0.47	**<0.001**
LCAT	Phosphatidylcholine-sterol acyltransferase	-0.33	**0.002**	0.37	**<0.001**	-0.22	**0.046**
IGLV743	Immunoglobulin lambda variable 7-43	0.32	**0.004**	-0.32	**0.004**	0.37	**<0.001**
SERPINA5	Plasma serine protease inhibitor	-0.37	**<0.001**	0.37	**<0.001**	-0.25	**0.022**
FABP1	Fatty acid-binding protein, liver	0.17	0.129	-0.11	0.322	0.29	**0.008**

HSP90AB1	Heat shock protein HSP 90-beta	-0.15	0.167	0.14	0.215	-0.32	**0.004**
P0DOX7	Immunoglobulin kappa light chain	0.33	**0.003**	-0.31	**0.005**	0.25	**0.027**
IGLL1	Immunoglobulin lambda-1 light chain	0.35	**0.001**	-0.29	**0.008**	0.24	**0.031**
**Table S3.** ***Cont.***							
IGL3	Immunoglobulin lambda constant 3	0.33	**0.003**	-0.29	**0.009**	0.26	**0.021**
IGFBP6	Insulin-like growth factor binding protein 6	-0.03	0.819	0.05	0.663	0.17	0.141
CPN2	Carboxypeptidase N subunit 2	-0.29	**0.007**	0.28	**0.013**	-0.32	**0.004**
MAN1A1	Mannosyl-oligosaccharide 1,2-alpha-mannosidase IA	-0.30	**0.006**	0.37	**<0.001**	-0.27	**0.016**
IST1	IST1 homolog	-0.25	**0.024**	0.30	**0.007**	-0.32	**0.004**
DSC2	Desmocollin-2	0.27	**0.017**	-0.25	**0.025**	0.22	0.052
GGT6	Glutathione hydrolase 6	-0.39	**<0.001**	0.46	**<0.001**	-0.18	**0.101**
PI16	Peptidase inhibitor 16	0.28	**0.010**	-0.31	**0.005**	0.36	**0.001**
FAM3C	Protein FAM3C	0.24	**0.026**	-0.19	**0.086**	0.30	**0.006**
SERPINI1	Neuroserpin	-0.25	**0.021**	0.38	**<0.001**	-0.44	**<0.001**
OMD	Osteomodulin	-0.15	0.193	0.16	0.149	-0.26	**0.017**
MMRN2	Multimerin-2	-0.16	0.144	0.23	**0.042**	-0.39	**<0.001**
VTA1	Vacuolar protein sorting-associated protein VTA1 homolog	-0.31	**0.006**	0.39	**<0.001**	-0.45	**<0.001**
SUSD2	Sushi domain-containing protein 2	-0.44	**<0.001**	0.53	**<0.001**	-0.18	0.096
TRFE	Selotransferrin	0.19	0.082	-0.20	0.071	0.67	**<0.001**

Table S4. Top 10 Reactome 2022 pathways of dysregulated proteins

**Pathway**	***p*-value**	***z*-score**
Hemostasis	0.000700	6.379
Intrinsic Pathway Of Fibrin Clot Formation	0.000722	58.31
Common Pathway Of Fibrin Clot Formation	0.000793	55.39
Keratan Sulfate Biosynthesis	0.001288	42.6
Regulation Of IGF Transport And Uptake By IGFBPs	0.001636	14.17
Platelet Degranulation	0.001714	13.94
Keratan Sulfate/Keratin Metabolism	0.001788	35.72
Response To Elevated Platelet Cytosolic Ca2+ R	0.001917	13.39
Gene And Protein Expression By JAK-STAT Signaling After Interleukin-12 Stimulation	0.002010	33.55
Formation Of Fibrin Clot (Clotting Cascade)	0.002245	31.63
